# Unusual Isolated Parapharyngeal Second Branchial Cleft Cyst: A Case Report and Literature Review

**DOI:** 10.1155/2020/8814071

**Published:** 2020-12-08

**Authors:** Emad A. Magdy, Geylan A. Fadali, Mahmoud Seif-Elnasr, Mohamed F. Fathalla

**Affiliations:** ^1^Department of Otorhinolaryngology-Head & Neck Surgery, Faculty of Medicine, Alexandria University, Alexandria, Egypt; ^2^Department of Pathology, Medical Research Institute, Alexandria University, Alexandria, Egypt

## Abstract

Second branchial cleft cysts (BCCs) are common congenital causes of neck swellings; however, isolated parapharyngeal space presentation is extremely rare, with only sporadic cases reported. Our objectives in this report are to describe a case and review different diagnostic and management strategies adopted in the current world literature. The case presented is a 26-year-old female with a large isolated parapharyngeal BCC extending to skull base in which first presenting symptoms were referred otalgia and painful side-to-side head rotation for months followed by odynophagia. A previously ordered computed tomography (CT) scan suspected a parapharyngeal abscess. Correct diagnosis was preoperatively achieved using magnetic resonance imaging (MRI) showing a 3.1 × 3.4 × 5.4 cm parapharyngeal BCC. Cyst was completely surgically excised transoral without complications. No evidence of recurrence has been noted after 24-month follow-up. A comprehensive world literature search for all reported cases in the last 30-years revealed thirty cases in 23 separate case reports with different diagnostic and surgical modalities adopted. Presentation and management strategies in such rare cases are discussed in detail. Our study shows that although rare, BCC diagnosis should be kept in mind while dealing with isolated parapharyngeal space swellings with MRI being key for successful preoperative diagnosis. If encountered, the transoral route can be a safe, aesthetically pleasing and effective way for complete surgical excision in contrast to most other parapharyngeal swellings, which are usually better excised via a transcervical approach.

## 1. Introduction

Branchial cleft cysts (BCCs) are congenital malformations arising from incomplete involution of branchial remnants. Around 90%–95% represent second branchial cleft anomalies that usually become clinically evident at ages 20 to 40 years [1–4].

Second BCCs are classified into four types according to Bailey-Proctor classification [5, 6]. *Type-I* cysts are situated along anterior border of sternocleidomastoid muscle beneath superficial cervical fascia. *Type-II* ones are the commonest and lie just laterally to great vessels beneath enveloping fascia of the neck. *Type-III* ones pass between internal and external carotid arteries. *Type-IV* cysts are situated in the pharyngeal mucosal space just deep to palatine tonsil and medial to great neck vessels, often extending upward towards skull base. Most second BCCs comprise the first three types, while type-IV cysts are extremely rare [4, 7, 8].

This report documents a large type-IV second BCC located in the parapharyngeal space presenting as a submucosal oropharyngeal swelling completely medial to great neck vessels. All reported cases in the world literature are reviewed, and different diagnostic and therapeutic modalities are discussed.

## 2. Case Presentation

A 26-year-old female presented with a 3-month history of right referred otalgia and pain on side-to-side head rotation. Sensation of throat fullness and odynophagia started later. Oropharyngeal examination showed a significant right smooth lateral pharyngeal wall submucosal swelling just behind the palatopharyngeal fold with normal overlying mucosa. Neck examination demonstrated no palpable neck swelling except two small tender right upper deep cervical lymph nodes (largest 2 × 1 cm). There was no fever or other signs of acute inflammation. Neurological symptoms were absent, and cranial nerve examination was normal. She had received broad-spectrum antibiotic and anti-inflammatory treatment for one week. Blood picture was normal with no leukocytosis.

**Figure 1:**
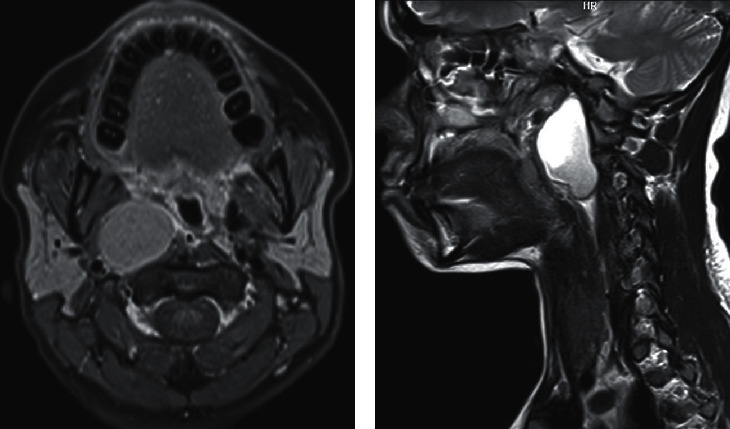
Preoperative magnetic resonance imaging. (a) Axial T1 postcontrast image with fat suppression showing the slightly hyperintense well-defined right parapharyngeal cyst with mild smooth linear wall enhancement and no enhancement of contents, medially indenting nasopharyngeal wall and displacing carotid sheath vessels posterolaterally. (b) Sagittal T2 sequence showing the well-defined cyst extending to skull base and contents heterogeneously hyperintense with fluid-fluid level.

The patient came with previously ordered contrast-enhanced computed tomography (CT) scan that showed a large right parapharyngeal cystic lesion with thin walls and mild mural enhancement. It averaged 3.1 × 3.4 × 5.4 cm in maximum anteroposterior, side-to-side, and craniocaudal dimensions, respectively. The lesion exerted a mass effect with indentation of nasopharyngeal/oropharyngeal walls and posterolateral displacement of right carotid sheath vessels with upper extension reaching skull base. Her preliminary imaging diagnosis of suspected right parapharyngeal space abscess was not entirely consistent with her clinical presentation; thus, we additionally ordered a neck magnetic resonance imaging (MRI). It revealed a well-defined right parapharyngeal cystic lesion with nearly same dimensions, slightly T1 hyperintense with contents showing no contrast enhancement and heterogeneously T2 hyperintense with fluid-fluid level, demonstrating restricted diffusion of contents (indicating high proteinaceous material) but not in cyst wall, suggesting a rare second BCC location (Figure 1).

Being a young female, the patient's request was to avoid any external approach if possible. After patient counseling, a decision of transoral surgical excision was taken with consent for transcervical incision if needed.

**Figure 2:**
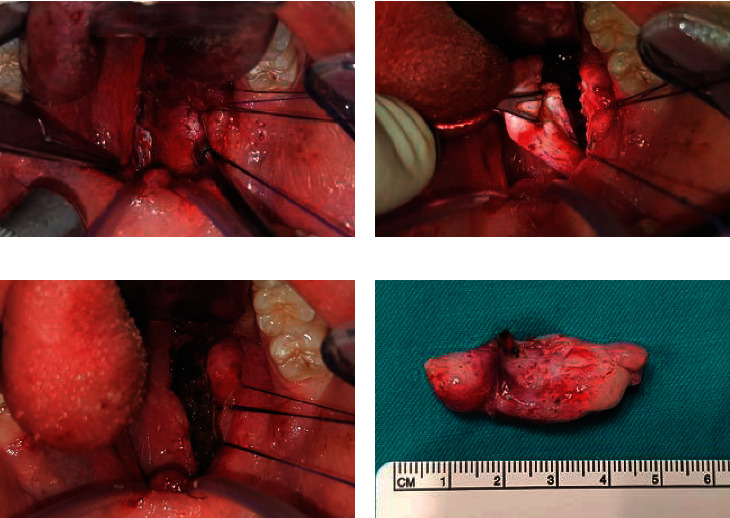
Intraoperative transoral excision views. (a) Vertical lateral pharyngeal wall incision exposing submucosal cyst. Soft palate catheter-retracted and ipsilateral tonsil suture-retracted to improve exposure. (b) Cyst wall grasped following planned evacuation of contents to facilitate lateral and superior blunt dissection off major neck neurovascular structures. (c) Resultant cavity filled with Fibrillar™ Surgicel® layers before pharyngeal incision is closed. (d) BCC surgical specimen after complete excision.

Under oral intubation general anesthesia with patient supine and neck extended, a Dingman mouth retractor was inserted. Adequate oropharyngeal exposure was achieved via soft palate catheter retraction and suture retraction of right tonsillar pillars. Surgery was performed under surgical loupe 3.5X visualization. Intraoperative aspiration revealed turbid golden-yellow fluid, which was sent for microbiological culture and sensitivity. A vertical mucosal incision overlying the oropharyngeal bulge was carried out with subsequent meticulous dissection over the cyst until its wall was identified. The large nature of the cyst required a deliberate evacuation of its contents for decompression to ease lateral and superior dissection from major neurovascular structures. Finally, the cyst wall was completely delivered from pharyngeal incision and sent for histopathological assessment. There was no evidence of an associated tract. Several layers of haemostatic Fibrillar™ Surgicel^®^ (Ethicon, LLC, San Lorenzo, Puerto Rico, USA) were applied to protect and obliterate the surgical cavity. Simple 4-0 Vicryl sutures were taken for pharyngeal incision closure (Figure 2).

**Figure 3:**
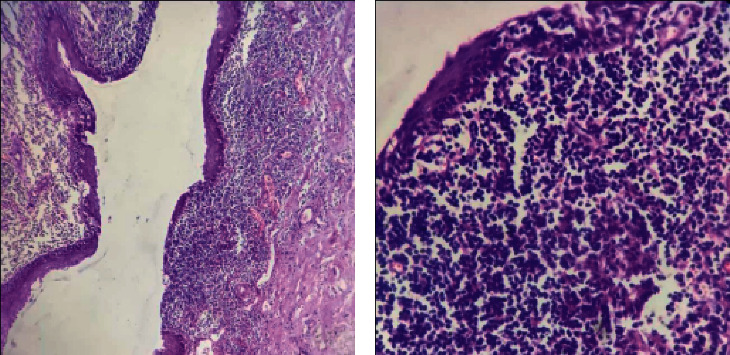
Histopathological examination of resected specimen. (a) Low power view of an invaginated part of the cyst, lined by attenuated stratified squamous epithelium with abundant subepithelial lymphoid tissue (H & E, ×100). (b) Close up view of a focally ulcerated area of the surface squamous epithelium encroached upon by heavy lymphocytic infiltrate including reactive lymphoid follicles (H & E, ×400).

**Figure 4:**
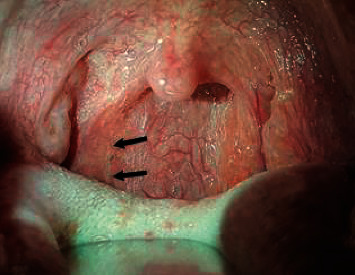
Postoperative (3-months) intraoral view showing complete incision healing (arrows).

**Figure 5:**
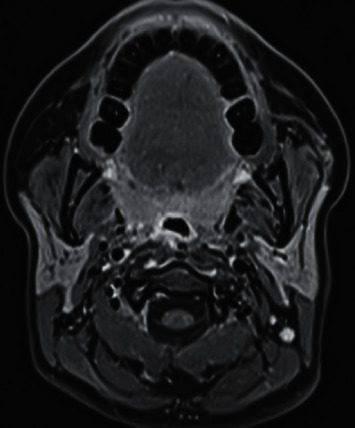
Postoperative (18-months) magnetic resonance imaging axial T1 postcontrast with fat suppression showing complete excision of right parapharyngeal cyst with no evidence of recurrence.

The patient was hospitalized for 48 hours postoperatively under broad-spectrum IV antibiotic coverage until oral feeding started. Early postoperative period was uneventful except for severe odynophagia that improved one week after surgery. No postoperative complications were encountered including wound hematoma or cranial nerve palsies. Final histopathological specimen examination confirmed BCC diagnosis (Figure 3). Microbiological aspirated fluid assessment yielded no growth excluding an active suppurative process. The patient has been followed-up for 24-months with no evidence of recurrence to date (Figures 4 and 5).

## 3. Discussion

The parapharyngeal space is a deep potential neck space having an inverted pyramid shape extending from skull base above to hyoid bone below [9]. It is classically divided into pre- and poststyloid parts. Generally, prestyloid lesions are usually salivary tumors displacing carotid sheath posterolaterally and poststyloid masses are more likely to be neurogenic in nature such as schwannomas and vascular neoplasms [10]. Isolated parapharyngeal type-IV second BCCs are exceptionally rare and usually described as case reports [11]. They expand predictably in path of least resistance in the soft tissue plane resulting in an oropharyngeal bulge.  Table 1 summarizes our PubMed and ScienceDirect search of reported cases in current world literature (starting 1989) in an attempt to compare our case to other reported cases regarding diagnostic and management strategies. Thirty cases were identified in 23 separate case reports found.

Although congenital in nature, type-IV cysts (as other BCCs) usually manifest in adulthood after gradually increasing in size following upper respiratory tract infections. Nevertheless, presentation age extremes were reported with Thaler et al.[16] having a 3-month infant and Howlett et al. [11] describing a 70-year-old case. Pediatric presentations are exceptional [16, 24, 27]. Symptoms are frequently insignificant and related to mass size with main presentation being sense of throat lump eventually leading to dysphagia or odynophagia without an external neck swelling [18, 20, 25, 32]. Unusually, Saussez et al. [29] reported a case with one-year history of snoring and no dysphagia. Recurrent deep neck abscess formation is another common alarming presentation leading to repeated aspiration and drainage procedures delaying diagnosis [18, 21, 31]. Severe infections might lead to multiple cranial nerve affection [14, 17, 23]. Interestingly, our case first symptom was referred otalgia and painful side-to-side neck mobility (probably from deep neck muscle irritation) of several months duration preceding odynophagia.

Cross-sectional imaging has become the mainstay diagnosis for BCCs. Radiologically, they can be distinguished from other lesions by their solitary, ovoid, sharply outlined, fluid-containing features [22]. CT with contrast enhancement is valuable in accurately delineating the cyst's location and extent. However, as our case demonstrates, MRI is considered the best diagnostic imaging modality for parapharyngeal space masses [15]. MRI BCC features include a well-marginated cystic mass that is CSF isointense on T1- and T2-weighted images with no significant postcontrast enhancement. Signal intensity may be increased on T1-images and show fluid-fluid level due to high protein content within the cyst. If infected, a thin rim of enhancement is possible. MRI is also superior in delineating the cyst's relation to great neck vessels and essential for differentiation from commoner parapharyngeal masses including deep lobe parotid lesions, parapharyngeal schwannomas with cystic degeneration, minor salivary gland tumors, and vascular neoplasms [9, 23].

After clinical and radiological diagnosis suspicion, BCC is confirmed histologically by a cystic cavity lined by epithelium and underlying connective tissue showing germinal centers. The lining is generally stratified squamous epithelium, and the germinal centers are contained within abundant lymphoid tissue. The connective tissue stroma underlying the epithelium contains areas of lymphoid tissue with reactive germinal centers but lacking true lymph node architecture as demonstrated in our case. [33–35].

Several conservative methods have been used in treatment of BCCs including repeated aspiration, incision and drainage, marsupialization and injection of sclerosant agents. All give only temporary relief and carry high incidence of recurrence [22, 28]. Consequently, complete surgical excision remains the main treatment. The route of surgical removal, however, should carefully consider morbidity from the surgical procedure itself. Two main approaches have been described in managing parapharyngeal BCCs, transcervical and transoral (Table 1). Each has its own pros and cons.

The transcervical route is considered the traditional approach for parapharyngeal masses [26]. Its obvious advantage is the wider operative field with better control on great neck vessels making dissection safer. It can include transparotid or transmandibular components allowing more access according to parapharyngeal mass location. Piccin et al. [10] reported a parapharyngeal BCC excised through a combined transcervical/transmandibular approach associated with tracheotomy. Although this provides the most complete exposure, associated morbidity in our opinion is unjustifiable. Ironically, the only complication in our world literature search was reported by Papay et al. [19] using the transcervical/transparotid approach for a parapharyngeal BCC with nasopharyngeal extension, which was followed by sympathetic chain and cranial nerve X and XII paresis postoperatively.

Transoral excision of a type-IV BCC was first described by Takimoto et al. [12] with the obvious advantage being esthetic. Another advantage is direct access to the medially located cyst avoiding major neck neurovascular dissections. The down side is poorer visibility and lack of vascular control on major neck vessels. Several authors proposed performing tonsillectomy to improve intraoral surgical access [11, 13, 31]. We found that using the Dingman mouth gag, tonsillar pillar retraction sutures, and soft palate catheter retraction all increases visibility and widens the access avoiding tonsillectomy morbidity. Intraoperative cyst fluid aspiration or even deliberate evacuation of its contents (as we did in our case) facilitates lateral and superior blunt dissection of cyst wall off major carotid sheath neurovascular structures, thus allowing complete safe resection of even large-sized cysts. Vidhyadharan et al. [30] in 2012 reported a single type-IV parapharyngeal BCC case in which transoral robotic resection was successfully achieved. Robotic surgical arms enabled safe tissue grasping and dissection. The ability of an assistant to introduce further surgical tools further facilitated intraoral excision. Unfortunately, robotic surgery is considered expensive and its use still limited to few medical centers in the world.

## 4. Conclusions

Isolated parapharyngeal BCC is extremely rare and hence is often misdiagnosed and treated as a deep neck abscess. Although congenital in origin, it mainly presents in adults with nonspecific symptoms. MRI is mandatory to differentiate from other more common parapharyngeal lesions. Complete surgical excision is the main line of treatment and most could be safely and effectively excised transoral unlike other parapharyngeal masses.

## Figures and Tables

**Table 1:** Type-IV second BCC cases reported in the world literature (since 1989).

Study (year)	Number of cases	Age (y), sex	Surgical approach	Complications
Takimoto et al. [12] (1989)	1	14, M	Transoral	
Dilkes et al. [13] (1990)	1	42, M	Tonsillectomy then transoral	
Gatot et al. [14] (1991)	2	27, M 19, M	Transcervical	XII CN palsy on presentation
Ruscito et al. [15] (1993)	1	27, F	Transoral	
Thaler et al. [16] (1993)	2	3 months, M 3, M	Transoral Transcervical	
Durrant et al. [17] (1994)	1	20, F	Transcervical	IX, X & XII CN palsies on presentation
Günerí et al. [18] (1994)	2	30, F 53, M	Transcervical/transparotid Transoral	
Papay et al. [19] (1994)	1	29, M	Transcervical/transparotid	Horner's syndrome, X & XII CN paresis PO
Chabot et al. [20] (1996)	2	22, F 17, F	Transoral Transcervical	
Paczona et al. [21] (1998)	2	38, F 34, M	Transoral Transcervical	
Bilgen et al. [22] (2001)	1	65, M	Transcervical	
Shin et al. [23] (2001)	1	35, F	Transcervical	IX, X & XII CN palsies on presentation
Choo et al. [24] (2002)	1	2, F	Not mentioned	
Gallego Aranda et al. [25] (2002)	1	34, M	Transcervical	
Dernis et al. [26] (2004)	1	29, M	Transcervical	
Ghosh et al. [27] (2006)	1	8, F	Combined transcervical/transoral	
Díaz-Manzano et al. [28] (2008)	1	40, M	Transoral	
Piccin et al. [10] (2008)	1	48, M	Combined transcervical/transmandibular	
Saussez et al. [29] (2009)	1	54, M	Transoral	
Vidhyadharan et al. [30] (2012)	1	56, M	Transoral robotic	
Gupta & Gupta. [9] (2013)	1	35, F	Transcervical	
Jung et al. [31] (2016)	3	51, M 57, F 46, M	Transoral ± tonsillectomy	
Howlett et al. [11] (2018)	1	70, M	Transoral + tonsillectomy	
Magdy et al. (2020) “present case”	1	26, F	Transoral	
Total	31	Age: range: 3 months–70 years (mean: 33.3 ± 18 years) Sex: M = 19/F = 12 (M: F ratio = 1.6 : 1)		

Abbreviations: Y, years; M, male; CN, cranial nerve; F, female; PO, postoperative.
